# Early and Intermediate-Term Outcome of Balloon Aortic Valvuloplasty in Children With Aortic Stenosis and Left Ventricular Dysfunction at Tertiary Care Hospital

**DOI:** 10.7759/cureus.8321

**Published:** 2020-05-27

**Authors:** Ram Chand, Abdul Sattar Shaikh, Naresh Kumar, Hussain Korejo, Arshad Sohail, Veena Kumari, Asif A Khan, Najma Patel

**Affiliations:** 1 Pediatric Cardiology, National Institute of Cardiovascular Diseases, Karachi, PAK; 2 Cardiology, National Institute of Cardiovascular Disease, Karachi, PAK; 3 Paediatric Cardiology, National Institute of Cardiovascular Diseases, Karachi, PAK; 4 Pediatric Cardiology, Rehman Medical Institute, Peshawar, PAK

**Keywords:** balloon aortic valvuloplasty, left ventricular dysfunction, aortic valve stenosis, ejection fraction, congestive cardiac failure, aortic insufficiency, aortic valve annulus, peak-peak pressure gradient, lv end-diastolic pressure

## Abstract

Background

Left ventricular (LV) dysfunction in patients with aortic valve stenosis (AVS) is seen in two scenarios: in neonates and in elderly patients. Neonatal AVS may present as a congestive cardiac failure (CCF), while older children rarely present with CCF if they have not been diagnosed early. Only a few reports of LV dysfunction with AVS have been described in the literature. However, there is a paucity of data regarding the safety and effectiveness of balloon aortic valvuloplasty (BAV) in children with AVS with LV dysfunction. Therefore, the aim of this study was to evaluate outcomes to establish the safety and effectiveness of BAV in children with AVS and LV dysfunction in improving LV function and survival.

Methods

A total of 160 BAVs were performed from 2004 to 2017; of these, 41 (25.6%) patients had LV dysfunction. We reviewed these cases, and data were obtained on clinical features, echocardiographic parameters including LV ejection fraction (LVEF) and LV dimensions, LV posterior wall, interventricular septal thickness, pressure gradient across the valve, aortic valve morphology and annulus and aortic insufficiency (AI), and angiographic parameters such as aortic and LV pressures, AI and annulus size, and balloon size. Echocardiography was done before the procedure, one day after intervention, at three months, at six months, and on regular follow-up. Mortality during and after the procedure and at follow-up was reported.

Results

Children who had undergone BAV for AVS and LV dysfunction within the age range of six to 192 months showed a significant reduction in peak-to-peak pressure gradient (PPG) from 73.5 ± 30 mmHg to 26.7 ± 6.7 mmHg and improvement in LVEF from 32.8 ± 11% to 54.3 ± 12.7% after 24 hours. Instantaneous gradient on echocardiography after three months showed PPG was 29.8 ± 7.7 mmHg and mean LVEF was 63 ± 8.6%. Mean LV end-diastolic pressure was 20.8 ± 4.7 mmHg and decreased to 13 ± 2.4 mmHg. Four patients died, all of whom had severe LV dysfunction - one died during the procedure and three died within six to 20 hours after successful BAV. On average follow-up of 6.4 ± 3.8 years, with a range of three months to 13 years, there was no mortality, pressure gradient increased to 40 ± 16.3 mmHg (range, 20 to 90 mmHg), and three had BAV after one, four, and six years, respectively. There was an increase in AI from mild to moderate in five patients, but they did not require any intervention. Four patients had aortic valve replacement (AVR) with two patients having an increase in pressure gradient and AI after eight and 13 years, respectively. One patient had AI (+3) after BAV had AVR after three years, and one patient who had a very thick and dysplastic aortic valve with LVEF of 20% and pulmonary hypertension (PH) had AVR after six months.

Conclusion

Patients with AVS who develop LV dysfunction deteriorate and die soon without treatment. Our data suggest that BAV in children with aortic stenosis and LV dysfunction is safe and effective in the normalization of LV function.

## Introduction

Aortic valve stenosis (AVS) accounts for 3% to 7% of all congenital heart diseases [1]. Left ventricular (LV) dysfunction in patients with AVS is seen in two scenarios: in neonates and in elderly patients. Neonatal AVS may present as a congestive cardiac failure (CCF) in the first few weeks of life. Older children rarely present with CCF if not diagnosed early. Patients with AVS who develop LV dysfunction have high mortality if left untreated. The onset of CCF is associated with an average survival of 1 to 1.5 years. AVS increases afterload, which inevitably leads to systolic and diastolic dysfunction [2]. This results in the adverse consequence of pressure overload of severe AVS [3]. Balloon aortic valvuloplasty (BAV) in children with AVS is considered as first-line palliative therapy [4]. Pure AVS results in compensatory ventricular hypertrophy proportional to the degree of obstruction. However, if an obstruction is not relieved early, some patients develop systolic dysfunction, which may either be due to an afterload mismatch or decrease in contractile function of the myocardium [5,6]. This is secondary to myocardial ischemia resulting from the combination of limited cardiac output, reduced coronary perfusion, and increased myocardial oxygen consumption leading to fibrosis in areas of the myocardium damaged by ischemia. LV adapts to the increased systolic pressure to maintain cardiac output through a hypertrophic process that involves the muscular and non-muscular elements [7]. Microscopically, hypertrophy along with interstitial fibrosis of LV is responsible for changes in systolic and diastolic functions, resulting in the reduction of stroke volume (SV) and compliance [8,9]. Clinically significant reduction of SV occurs in 51% of patients even with low-grade aortic stenosis [10]. The LV systolic function, expressed by LV ejection fraction (LVEF), is maintained if the increased wall thickness is sufficient to counteract the elevated systolic pressure intracavitary [11]. Optimal LV diastolic function depends on the LV compliance in diastole, which enables the LV to fill from low left atrial pressure [12]. Only a few studies of aortic valvoplasty in patients with aortic stenosis and LV dysfunction in children are published, but no local data are available. The aim of this study was to evaluate the early and intermediate outcomes to establish the safety and effectiveness of BAV in children with aortic stenosis and LV dysfunction in improving LV function and survival.

## Materials and methods

**Figure 1:**
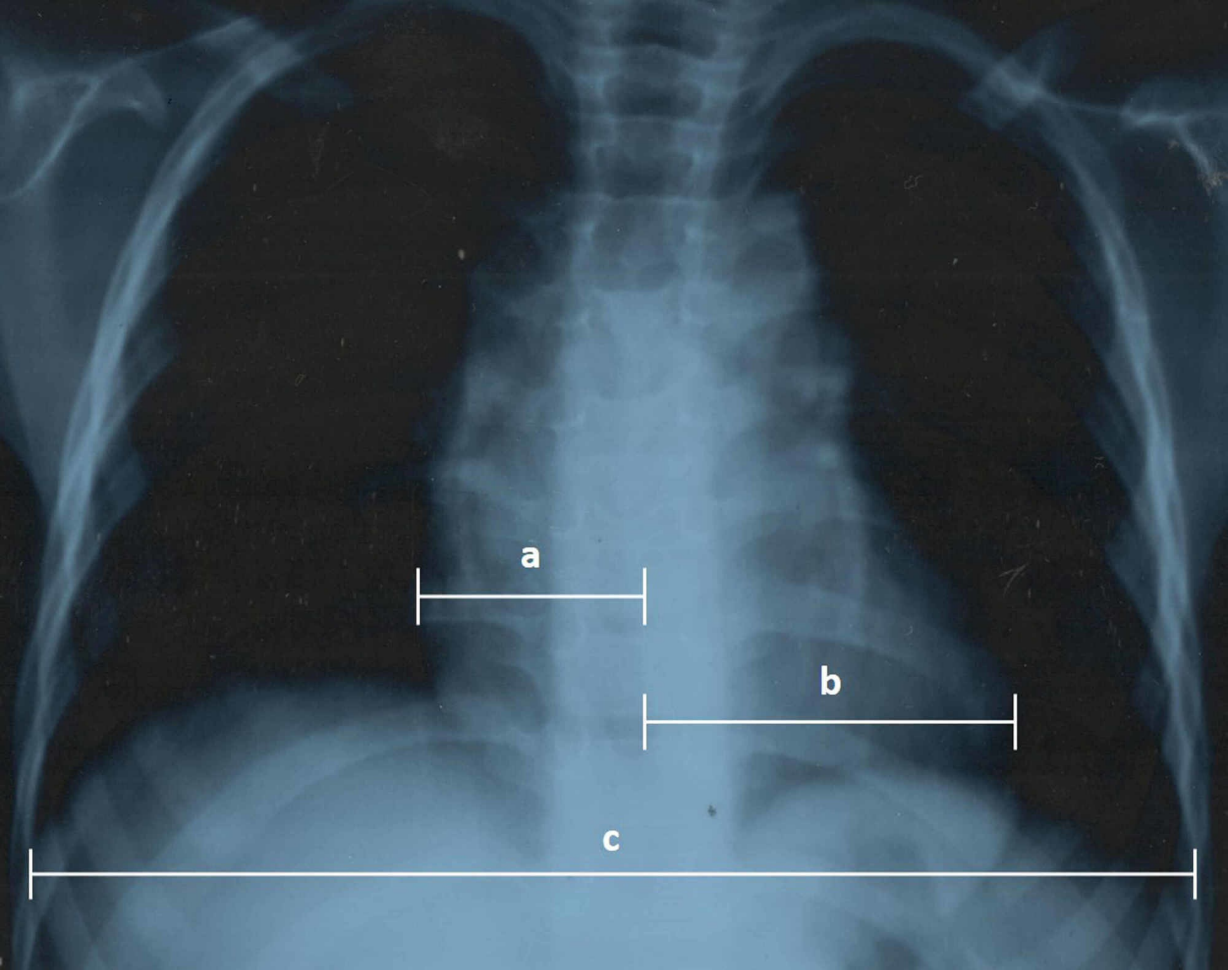
Chest X-ray posterior-anterior view showing cardiomegaly Cardiothoracic ratio = (a + b)/c

From 2004 to 2017, 160 children ages 15 days to 16 years had BAV at the Pediatric Cardiology Department of the National Institute of Cardiovascular Diseases in Karachi, Pakistan. Of these, 41 (25.6%) patients had LV dysfunction. For this retrospective study, we reviewed the record of these patients, ages six months to 192 months. Infants younger than six months with AVS and LV dysfunction were excluded to rule out the continuum of critical AVS. Weight ranged from 4.5 to 50 kilograms. Patients were categorized as severe, moderate, and mild LV dysfunction against criteria of LVEF ≤35%, 36% to 45%, and 45 to 55%, respectively. After approval from the ethical review committee, data were obtained based on their clinical features, echocardiographic, and angiographic parameters. All patients were symptomatic with a history of poor feeding, syncopal attacks, dyspnea on exertion, chest pain, and hypotensive with blood pressure less than one-third percentile. Chest X-rays had a cardiothoracic ratio of 0.68 (0.6-0.75), chest X-ray posterior-anterior view showing cardiomegaly is presented in Figure 1.

**Figure 2:**
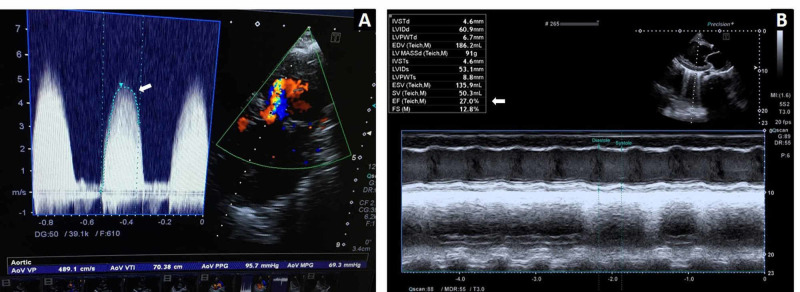
Color Doppler echocardiography by continuous-wave tracing in suprasternal view showing pressure gradient across aortic valve (A) and parasternal long-axis view showing left ventricular dimension and ejection fraction (B)

All echocardiographs were performed by pediatric cardiologists. Echocardiographic parameters were noted as morphology (unicuspid, bicuspid, tricuspid) of the aortic valve, aortic valve annulus (AVA), aortic insufficiency (AI) (if any), LV function, LV dimensions, LV posterior wall, and interventricular septal thickness. Instantaneous pressure gradient peak and mean were recorded by continuous-wave Doppler tracing in multiple planes, including apical five-chamber, apical three-chamber, right parasternal, subxiphoid long axis, and suprasternal views, and maximum gradients were considered, as shown in Figure 2.

**Figure 3:**
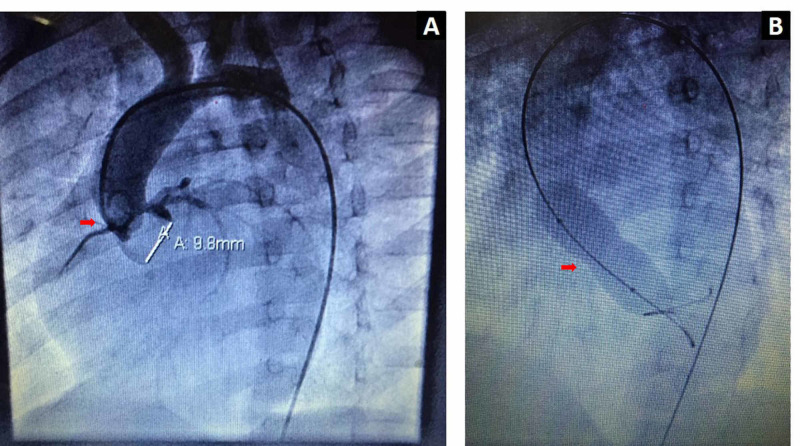
Aortic root angiogram in the left anterior oblique view showing aortic valve annulus (A) and balloon dilatation (B)

The severity of AI was classified as none, trivial, mild, moderate, and severe or graded from 0 to IV [13]. Angiographic parameters were also noted as aortic and LV pressures, AVA size, balloon size, and degree of AI (if any). Echocardiography was done before the procedure, one day after intervention, at three months, and on regular follow-up. The procedure was performed under general anesthesia in 25 patients and under conscious sedation with local anesthesia in 16 patients. A retrograde approach was used in all patients. Heparin in a dose of 70 to 100 IU/kg/IV was administered during the procedure immediately after the arterial assessment. BAV was performed via bi-plane fluoroscopy. Aortic pressure was recorded and an aortic root angiogram in the left anterior oblique view was done for AVA and AI (if any), as shown in Figure 3A. A soft tip guidewire was used to pass the catheter via a stenotic aortic valve into the LV. Then, a peak-to-peak pressure gradient (PPG) was calculated by using peak aortic and LV pressures. A balloon diameter of 70% of the annulus was first chosen and then gradually increased up to 90% of the annulus if the desired results were not obtained (Figure 3B).

Before changing the balloon size, the AI was checked by aortogram. Following angiography, a further intervention was not performed if an AI of plus 1 or greater was detected or if the balloon/annulus ratio was 1. After the procedure, PPG and LV end-diastolic pressure (LVEDP) were also measured. A reduction of the PPG of at least 50% or a residual gradient of less than 25 mmHg were defined as satisfactory results.

Collected data were entered and analyzed using IBM Statistical Package for the Software Sciences Statistics for Windows, Version 21.0. (IBM Corp., Armonk, NY). Appropriate mean ± SD or median (interquartile range) were calculated. An appropriate t-test or Mann-Whitney U test was applied. The cutoff p-value for statistical significance was ≤ 0.05.

## Results

Forty-one patients with LV dysfunction were included. Ages ranged from six to 192 months, and weight ranged from 4.5 to 50 kg. Male patients and female patients were 61% and 39%, respectively. A history of poor feeding was found in 26.8%, exertional dyspnea in 53.7%, syncopal attack in 12.2%, and chest pain in 7.3% (Table 1).

**Table 1:** Demographic characteristics and clinical presentations Max = maximum, Min = minimum

Characteristics	Total
N	41
Gender	
Male	61% (25)
Female	39% (16)
Age (months)	
Mean ± SD	77.48 ± 47.65
Max – Min	192 – 6
Weight (kg)	
Mean ± SD	15.2 ± 10.5
Max – Min	50 – 4.5
Clinical presentation	
Poor feeding/lethargy	26.8% (11)
Exertional dyspnea	53.7% (22)
Syncopal attack	12.2% (5)
Chest pain	7.3% (3)

Echocardiographic data obtained before BAV. Pre-dilatation mean peak instantaneous pressure gradient was 73.5 ± 30 mmHg (range, 20 to 140 mmHg). LVEF was 32.8 ± 11% (range, 15% to 55%) with severe, moderate, and mild LV dysfunction of 58.5%, 29.3%, and 12.2%, respectively. Pre-dilatation AI of grade 0 was found in 80.5% and grade 1 in 19.5% of patients (Table 2).

**Table 2:** Echocardiographic results before balloon aortic valvuloplasty Max = maximum, Min = minimum

Characteristics	Total
N	41
Peak pressure gradient	
Mean ± SD	73.5 ± 30 mmHg
Max – Min	140 - 20 mmHg
Valve morphology	
Unicuspid	0% (0)
Bicuspid	36.6% (15)
Tricuspid	63.4% (26)
Ejection fraction (%)	
Mean ± SD	32.8% ± 11
Max – Min	55 - 15%
Left ventricular dysfunction	
Severe (ejection fraction ≤ 35%)	58.5% (24)
Moderate (ejection fraction 36-45%)	29.3% (12)
Mild (ejection fraction 46-55%)	12.2% (5)
Aortic insufficiency	
Grade 0	80.5% (33)
Grade I	19.5% (8)

After successful BAV, there was a significant reduction in PPG from 73.5 ± 30 mmHg to 26.7 ± 6.7 mmHg (range, 16 to 44 mmHg) with a p-value of <0.001 in all patients. LVEDP also decreased from mean 20.8 ± 4.7 mmHg to 13 ± 2.4 mmHg. After successful BAV, approximately 9.7% of patients had an AI of grade 3 to 4. All patients had a femoral artery assessed, and none of the patients developed vascular complications. In our study, in-hospital mortality was found in four (9.8%) patients, all of whom had severe LV dysfunction. One six-month-old patient (2.4%) who died during the procedure had an EF of 20% and intractable heart failure. Three patients (7.3%) died six to 20 hours after successful BAV; two had mild AI, and one had no AI. All were stable and they died suddenly. In our study, 14 patients (34.1%) had rhythm disturbance (premature ventricular contractions) with no sustained ventricular tachycardia noted, and two patients (4.9%) developed bradycardia (temporarily), which resolved at the end of the procedure (Table 3).

**Table 3:** Intra-procedural characteristics and post-procedure characteristics LVEDP = left ventricular end diastolic pressure, Max = maximum, Min = minimum, PVCs = premature ventricular contractions

Characteristics	Total
N	41
Balloon/annulus ratio	
Mean ± SD	0.8 ± 0.06
Max – Min	0.9 - 0.7
Number of balloon inflation	
Median (Max – Min)	3 (4 – 1)
LVEDP before balloon dilatation	
Mean ± SD	20.8 ± 4.7 mmHg
Max – Min	30 – 14 mmHg
LVEDP after balloon dilatation	
Mean ± SD	13 ± 2.4 mmHg
Max – Min	19 – 7 mmHg
Aortic insufficiency	
Grade 0	24.4% (10)
Grade I	31.7% (13)
Grade II	34.1% (14)
Grade III	7.3% (3)
Grade IV	2.4% (1)
Post-procedure complications	
Bradycardia (temporary)	4.9% (2)
Rhythm disturbance (PVCs)	34.1% (14)
Mortality during the procedure	2.4% (1)
Mortality within 6 to 24 hours of the procedure	7.3% (3)

Instantaneous pressure gradient on echocardiography after three months was also noted as 29.8 ± 7.7 mmHg with a p-value of < 0.001. All showed an increase in LVEF from 32.8 ± 11% to 54.3 ± 12.7% (range, 20% to 78%) after 24 hours (p < 0.001) and to mean LVEF 63% ± 8.6 (range, 30% to 76%) after three months (p < 0.001; Table 4). One patient who had an LVEF of 15% before dilatation had an LVEF of 30% after dilatation and was kept on dobutamine infusion for one month; however, his LVEF improved, but he had moderate LV dysfunction with an LVEF of 40% after six months of BAV. All had LVEF of more than 60% except for two patients.

**Table 4:** Echocardiographic assessment after balloon aortic valvuloplasty and at follow-up Max = maximum, Min = minimum

Characteristics	Baseline	After 24 hours	After three months
N	41	37	37
Peak pressure gradient			
Mean ± SD	73.5 ± 30 mmHg	26.7 ± 6.7 mmHg	29.8 ± 7.7 mmHg
Max – Min	140 – 20 mmHg	44 – 16 mmHg	46 – 16 mmHg
P-value as compared to baseline		<0.001	<0.001
Ejection fraction (%)			
Mean ± SD	32.8 ± 11%	54.3 ± 12.7%	63 ± 8.6%
Max – Min	55 – 15%	78 – 20%	76 – 30%
P-value as compared to baseline		<0.001	<0.001

On an average follow-up of 6.4 ± 3.8 years, with a range of three months to 13 years, there was no mortality, and pressure gradient increased to 40 ± 16.3 mmHg (range, 20 to 90 mmHg). Three patients had BAV after one, four, and six years, respectively. There was an increase in AI from mild to moderate in five patients, but they did not require any intervention. Four patients had aortic valve replacement (AVR) with two patients having an increase in gradient and AI after eight and 15 years, respectively. One patient had AI (+3) after BAV had AVR after three years, and one patient who had a very thick and dysplastic aortic valve with LVEF of 20% and pulmonary hypertension (PH) had AVR after six months.

## Discussion

In patients with AVS, diastolic dysfunction is common due to hypertrophied noncompliant LV; however, systolic dysfunction beyond the neonatal period and early infancy is not common. LV dysfunction in this condition may be due to subendocardial contractile dysfunction caused by long-standing high wall stress or diffuse myocardial fibrosis, scarring, apoptosis, or it could be a “pseudo LV dysfunction” (i.e., simple mechanical stunning due to high afterload). Severe pressure overload in AVS resulting in LV systolic dysfunction is an undesirable consequence [14]. In our study, 25.6% of patients with AVS had LV dysfunction, perhaps due to late diagnosis or referrals. We also found decreased PPG, improved LV functions, and decreased LVEDP after successful BAV. This reduction in pressure gradient and improvement in LVEF was reflected in transthoracic echocardiography, which was done one day after intervention, at three months, and on regular follow-up. Little data are available in children of aortic valvuloplasty in this age group of patients; however, in adults, several reports of AVR in patients with LV dysfunction are present. After BAV, patients who developed AI had been identified previously as the main indication for aortic valve surgery [15]. The balloon/annulus ratio also showed a significant correlation with the degree of AI. However, a significantly low incidence of AI in our study is due to the use of a smaller balloon/annulus ratio with mean ± SD (0.8 ± 0.06). It was present as trivial in 31.7%, mild in 34.1%, moderate in 7.3%, and severe in 2.4% of cases. Reich and colleagues showed that an independent risk factor for AI after BAV is the functionally bicuspid aortic valve morphology [16]. In-hospital mortality was found in 9.8% of patients. The retrograde femoral artery approach is used routinely in most centers [17], and it was also used in our study. None of our patients developed vascular complications, although these patients were vulnerable to high risk during the procedure. Ventricular ectopic beats are common during the wire placement and catheter exchanges, and these may disintegrate into ventricular tachycardia or ventricular fibrillation because of dilated LV. However, sinus bradycardia was noted in one to two minutes, which was then followed by asystole. There was an increase in transvalvular gradient during follow-up; however, none of the patients required reintervention. There were no late deaths noted.

Our study was a single-center retrospective study of prospectively collected data and had all the inherent limitations of a retrospective design. Secondly, follow-up data were based on voluntarily follow-up patient visits on a given date, and the duration of follow-ups was not the same for all patients.

## Conclusions

Patients with AVS who develop LV dysfunction deteriorate very soon, if they do not receive early intervention, resulting in poor outcomes. Data suggest that transcatheter BAV in children with aortic stenosis and LV dysfunction are associated with a significant improvement in LV function and a reduction in PPG. BAV can be a better alternative to the surgical correction of AVS.
